# Facile Synthesis of Novel Conducting Copolymers Based on N-Furfuryl Pyrrole and 3,4-Ethylenedioxythiophene with Enhanced Optoelectrochemical Performances Towards Electrochromic Application

**DOI:** 10.3390/molecules30010042

**Published:** 2024-12-26

**Authors:** Huixian Li, Xiaomeng Sun, Datai Liu, Xinchang Liu, Xianchao Du, Shuai Li, Xiaojing Xing, Xinfeng Cheng, Dongqin Bi, Dongfang Qiu

**Affiliations:** College of Chemistry and Pharmaceutical Engineering, Nanyang Normal University, Nanyang 473061, China

**Keywords:** electrochemical polymerization, polypyrrole, 3,4-ethylenedioxythiophene, conducting polymer, electrochromic

## Abstract

In this article, a series of novel conducting copolymers P(FuPy-*co*-EDOT) are prepared via cyclic voltammetry electropolymerization method by using N-furfuryl pyrrole (FuPy) and 3,4-ethylenedioxythiophene (EDOT) as comonomers. The molecular structure, surface morphology, electrochemical, and optical properties of the resulting copolymers are characterized in detail upon varying the feed ratios of FuPy/EDOT in the range of 1/1 to 1/9. The results demonstrate that the prepared P(FuPy-*co*-EDOT) copolymers with a higher proportion of EDOT units (FuPy/EDOT: 2/8~1/9) possess good redox activity, tunable optical absorption performances, and low band gaps (1.75~1.86 eV). Spectroelectrochemistry studies indicate that the resulting copolymers with increased EDOT units show strengthened electrochromic characteristics, exhibiting a red-to-green-to-blue multicolor reversible transition, especially for the P(FuPy_1_-*co*-EDOT_9_) copolymer films. They also show increased optical contrast (9~34%), fast response time (0.8~2.4 s), and good coloring efficiency (110~362 cm^2^ C^−1^). Additionally, the complementary bilayer P(FuPy-*co*-EDOT)/PEDOT electrochromic devices (ECDs) are also assembled and evaluated to hold excellent electrochromic switching performances with relatively high optical contrast (25%), rapid response time (0.9 s), and satisfactory coloring efficiency (416 cm^2^ C^−1^). Together with the superior open circuit memory and cycling stability, they can be used as a new type of electrochromic material and have considerable prospects as promising candidates for electrochromic devices.

## 1. Introduction

Electrochromism is defined as the reversible alteration in color exhibited by materials when subjected to an external electric field or current [1,2,3]. Electrochromic materials offer several advantages, including high color contrast, rapid response times, and low operating voltages, rendering them highly applicable in various domains such as smart windows, antiglare rearview mirrors, and display technologies [4,5,6,7,8,9]. The primary categories of electrochromic materials encompass inorganic metal oxides, organic small molecules, organometallic complexes, and conducting polymers [10,11,12,13,14]. Among various conducting polymers, polypyrrole (PPy), as a conjugated heterocyclic conductive polymer, exhibits advantages such as ease of synthesis, high conductivity, good electrochemical activity, and electrochromic properties, showing considerable application potential in electrochemical energy storage, sensors, corrosion resistance, and electrochromism [15,16,17,18,19]. However, the rigid molecular structure of polypyrrole leads to difficulties in processing, poor adhesion to substrates, limited color variation, and inadequate cycling stability, which may hinder its application in the electrochromic field. Consequently, various approaches, such as structural modification, copolymerization, dye doping, and material composites, were employed to improve the optoelectrochemical performances of PPy-based electrochromic materials [20,21,22,23,24,25].

Recently, significant advancements have been made in the development of conductive polymer electrochromic materials based on polypyrrole derivatives. This progress has been achieved through the modification of the molecular structure of pyrrole monomers, followed by electrochemical polymerization or copolymerization with other monomers [26,27,28,29]. Extensive studies have focused on modifying the structure of polypyrrole by introducing aromatic rings (e.g., phenyl derivatives) or heteroaromatic rings (e.g., thiophene, carbazole, benzothiadiazole) into the main or side chains of polypyrroles. This is achieved through *N*-substitution, α- or β-substitution, and donor-acceptor strategies, with the aim of enhancing their optoelectrochemical performances [30,31,32]. The π-π conjugation effect between adjacent component units has led to the design and synthesis of a series of polypyrrole derivatives with diverse optical absorption, electrochemical activity, and electrochromic properties. Among these, the 2,5-dithienyl-1-substituted-pyrrole (SNS) -based polypyrrole derivatives have garnered significant attention due to their exceptional optoelectronic properties [33,34,35]. These polymers not only possess the common advantages of polythiophene and polypyrrole, such as low oxidation potential, low band gap, and good conductivity, but also benefit from the modifiable properties from the substituents at N position of the pyrroles. By introducing various functional groups at the N position of pyrroles (e.g., alkyl chains, substituted aromatics with electron-donating or electron-withdrawing groups, aromatic heterocycles, and electroactive ferrocene), the dissolution performance, charge transfer, thermal stability, optical properties (e.g., absorption and emission), and photoelectrochemical properties of them can be regulated [16,36,37]. This further explores and expands their potential applications in electrochemical energy storage materials, light-emitting diodes, and electrochromic devices.

In addition, electrochemical copolymerization is one of the essential methods for modulating the optoelectrochemical performances of the PPy conducting polymers [36]. This approach has proven to be simple and effective. By copolymerizing different monomers, it is possible to obtain copolymers with properties that are entirely distinct from those of the corresponding homopolymers. These copolymers not only possess the characteristics of the individual monomer units but may also exhibit synergistic effects, leading to the emergence of new properties. Especially, as a typical electron-donating π-conjugated monomer, 3,4-ethylenedioxythiophene (EDOT) possesses a unique molecular coplanarity ability that endows it with excellent electrochemical activity. This makes it frequently used as a comonomer to prepare various polypyrrole-based conducting copolymers with enhanced electrochemical and spectroelectrochemical performances [38,39,40,41].

To date, though numerous studies have documented electrochromic materials derived from *N*-substituted polypyrroles, there is a paucity of research focusing on *N*-furfuryl modified polypyrrole derivatives. Brovelli et al. exclusively synthesized a conducting polymer based on 1-furfurylpyrrole through electrochemical polymerization, focusing on its carrier injection mechanism [42]. Nevertheless, investigations into its spectroelectrochemistry and electrochromic properties remain scarce to date. It is of interest to extensively understand the structure and optoelectronic properties relationships of this type of polypyrrole derivatives with side furfuryl groups. Moreover, in light of the facile and efficient modification method of copolymerization, we were also curious to explore if the combination of *N*-furfuryl pyrrole derivatives with EDOT units would result in copolymers with improved electrochemical and electrochromic performances.

In this study, *N*-furfuryl pyrrole (FuPy) and 3,4-ethylenedioxythiophene (EDOT) were employed as comonomers to design and synthesize a series of copolymers, denoted as P(FuPy-*co*-EDOT), through cyclic voltammetry. Through fine-tuning of the comonomer ratios, an extensive investigation was undertaken to evaluate the structural morphology, electrochemistry, optoelectronic properties, and electrochromic switching performances of the synthesized copolymer films as well as their solid-state ECDs.

## 2. Results and Discussion

### 2.1. Electrochemical Polymerization

**Figure 1:**
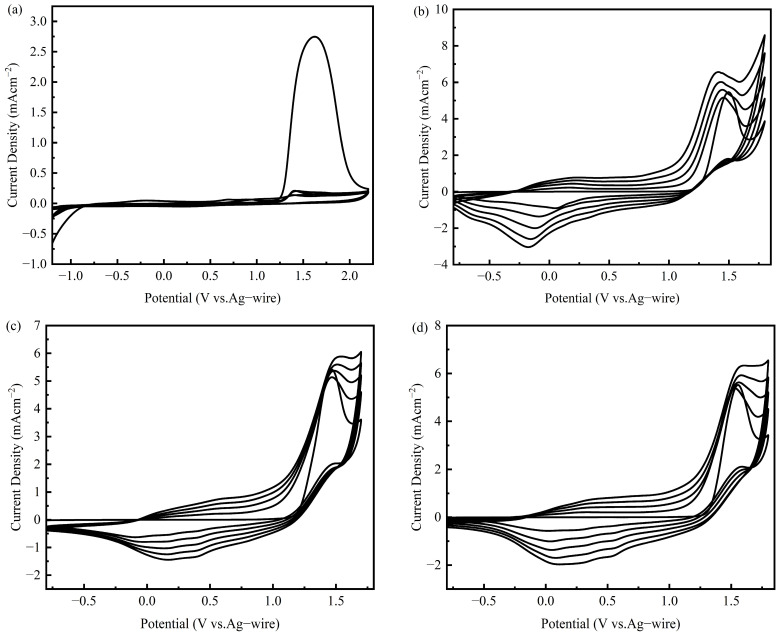
Cyclic voltammogram curves of the monomers in 0.1 mol/L Bu_4_NClO_4_/ACN electrolyte solution with a scan rate at 100 mV s^−1^: (**a**) 5 mM FuPy; (**b**) 5 mM EDOT; (**c**) 5 mM FuPy/EDOT (molar ratio: 2/8); (**d**) 5 mM FuPy/EDOT (molar ratio: 1/9).

The electrochemical polymerization behaviors of the monomers FuPy, EDOT, and their mixtures were investigated using the cyclic voltammetry method in 0.1 mol/L Bu_4_NClO_4_/ACN electrolyte solution, employing a platinum wire as the working electrode, another platinum wire as the counter electrode, and a silver wire as the reference electrode. The CV curves of the monomer FuPy show that the oxidation peak current at 1.72 V of the monomer is highest during the first scan, followed by a sharp decrease from the second scan (Figure 1a). This unusual phenomenon was reported by F. Brovelli et al. [42]. Combining their previous studies about electrochemical behavior and density functional theory (DFT) calculations, it has been suggested that the electrochemical oxidative polymerization of the FuPy monomer might mostly occur at the pyrrole rings, although there was the possibility of oxidation of the furan rings. As the scan progressed, a thin film of homopolymer PFuPy was formed on the Pt electrode surface, while for the monomer EDOT (Figure 1b), it showed an obvious oxidation peak from the monomer around 1.50 V, accompanied by a reduction peak (*E*_pc_) around −0.20 V as the scan continued. The peak currents (i.e., *E*_pa_ and *E*_pc_) increased with the number of cycles, and a relatively thicker polymer film of PEDOT was observed on the electrode surface. It is generally known that successful electrochemical copolymerization of different monomers typically occurs when their onset oxidation potentials are relatively close to each other [37]. Notably, the onset oxidation potentials (*E*_onset_) of both the FuPy and EDOT monomers were determined to be 1.32 and 1.27 V, respectively, indicating their close values and suggesting the feasibility of electrochemical copolymerization.

Furthermore, through adjusting the molar ratios of the mixtures of FuPy/EDOT from 1/1, 3/7, 2/8, to 1/9, the electrochemical copolymerization behaviors between FuPy and EDOT were also studied. As for the FuPy/EDOT mixtures at the ratio of 1/1, the CV curves showed a similar behavior to that of FuPy with an oxidation peak located at 1.65 V in the first scan (Figure S1a). When the ratio of FuPy/EDOT decreased to 3/7, it showed an oxidation peak located at 1.62 V in the first segment, which may be attributed to the introduction of more monomer EDOT (Figure S1b). As the CV scans continued, both of them (i.e., FuPy/EDOT: 1/1, 3/7) underwent a similar trend to that of pure FuPy monomers. When further decreasing the molar ratio of FuPy/EDOT to 2/8, apart from an oxidation peak at 1.45 V in the first segment, it is exciting to see that the redox current increased in the range of 0 V to 0.50 V as the sweep proceeded (Figure 1c). Upon further adjustment of the ratio of FuPy/EDOT to 1/9, it exhibited a similar and improved electropolymerization behavior to the former feed ratio of 2/8, demonstrating a remarkable increase in redox current between 0 V and 0.50 V (Figure 1d).

**Scheme 1:**
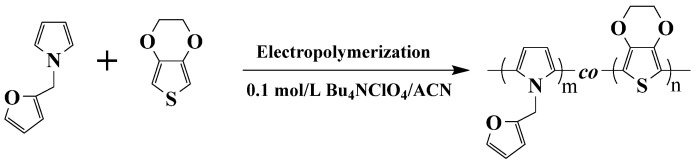
Synthetic route of the P(FuPy-*co*-EDOT) copolymers based on the FuPy and EDOT comonomers.

Based on the above features, it is clear to see that the redox pairs gradually shifted to lower voltages when the ratios of EDOT increased. This may be due to the fact that the incorporation of EDOT comonomers could improve the electrochemical oxidative coupling reaction of the both FuPy and EDOT monomers, facilitating the polymerization to produce conducting copolymers with high conjugation extent. Accordingly, the electrochemical copolymerization route for the FuPy and EDOT comonomers was proposed, as illustrated in Scheme 1. Here, P(FuPy_2_-*co*-EDOT_8_) and P(FuPy_1_-*co*-EDOT_9_) were used to denote the corresponding copolymers prepared with feed ratios of FuPy/EDOT in 2/8 and 1/9, respectively. The above studies demonstrated that by rational adjustment of the FuPy/EDOT comonomer feed ratios, the P(FuPy-*co*-EDOT) copolymers based on FuPy and EDOT were successfully obtained through electrochemical polymerization.

### 2.2. FT-IR Spectra Characterizations of the P(FuPy-co-EDOT) Copolymers

**Figure 2:**
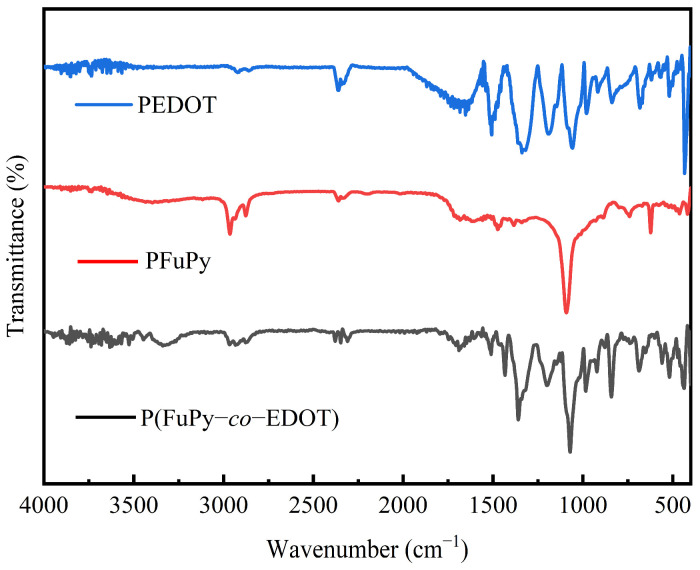
The FT-IR spectra of the PFuPy and PEDOT homopolymers, as well as the copolymer P(FuPy-*co*-EDOT).

The molecular structures of the obtained P(FuPy-*co*-EDOT) copolymers were characterized using FT-IR spectroscopy analysis. Here, the P(FuPy_2_-*co*-EDOT_8_) copolymer was chosen as an example for FT-IR testing, and was further compared with that of the PFuPy and PEDOT homopolymers (Figure 2).

In comparison to the infrared characteristic peaks of FuPy and EDOT monomers (Figure S2), the PFuPy homopolymer displayed peaks at 1473 cm^−1^ and 1383 cm^−1^, corresponding to the stretching vibrations of the C=C bond and C-N bond, respectively. The band at 1091 cm^−1^ is assigned to the C-O-C bond stretching in the furan ring, and the out-of-plane deformation of the C-H bond in the pyrrole ring appears at 741 cm^−1^ [43]. Notably, this peak nearly disappeared in the spectra of both the homopolymer PFuPy and the copolymer P(FuPy_2_-*co*-EDOT_8_), suggesting that polymerization predominantly occurred at the pyrrole ring. For the PEDOT homopolymer film, peaks at 1192 cm^−1^ and 1058 cm^−1^ are attributed to the C-O-C bond stretching in the EDOT ring. The stretching vibrations of the C=C and C-C bonds appear at 1506 cm^−1^ and 1338 cm^−1^, respectively, while bands at 682 cm^−1^ and 980 cm^−1^ correspond to the C-S-C stretching vibrations of thiophene ring [44]. The characteristic band of the EDOT monomer at 3111 cm^−1^, related to the aromatic ring C-H bond stretching, almost disappeared in the spectra of both the homopolymer PEDOT and the copolymer P(FuPy_2_-*co*-EDOT_8_), indicating polymerization at the 2 and 5 positions of the EDOT thiophene ring. In the infrared spectrum of P(FuPy_2_-*co*-EDOT_8_), characteristic peaks corresponding to both PFuPy and PEDOT homopolymers are clearly identifiable. The stretching vibrations of C-N, C-O-C, C=C, C-C, and C-S-C are observed at 1359 cm^−1^, 1068 cm^−1^, 1435 cm^−1^, 1361 cm^−1^, 692 cm^−1^, and 987 cm^−1^, respectively. The above features further confirmed the successful polymerization of the FuPy and EDOT comonomers to afford the corresponding P(FuPy-*co*-EDOT) copolymer film.

### 2.3. Surface Morphology of the P(FuPy-co-EDOT) Copolymer Films

**Figure 3:**
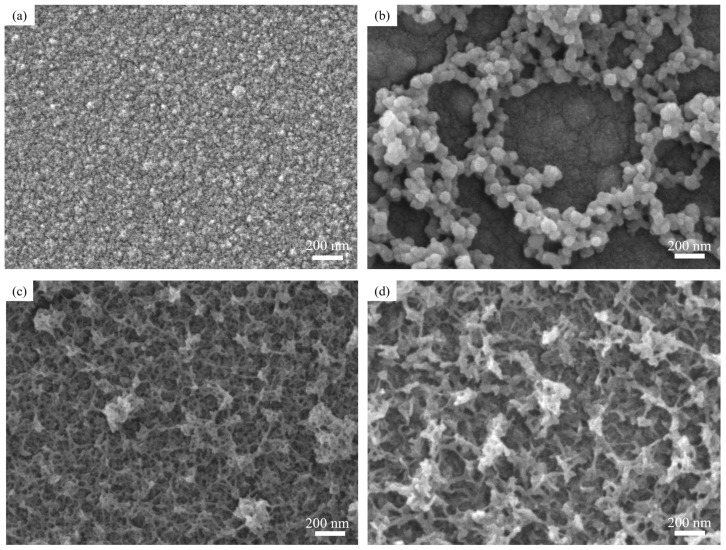
The SEM images of the polymer films (scale bar: 200 nm): (**a**) PFuPy; (**b**) P(FuPy_2_-*co*-EDOT_8_); (**c**) P(FuPy_1_-*co*-EDOT_9_); (**d**) PEDOT.

The micromorphologies of the resulting polymer films, including the homopolymers and copolymers, were analyzed via the SEM technique. All these films were dedoped at −0.8 V for about 100 s using the potentiostatic method, rinsed with ACN solvent, and dried under ambient conditions prior to testing. The PFuPy film exhibited a dense stacking of particles with a flat surface, as shown in Figure 3a. Similarly, the P(FuPy_3_-*co*-EDOT_7_) copolymer film also presented a relative flat and densely packed surface (Figure S3), while for P(FuPy_2_-*co*-EDOT_8_), there were some particle stacking clusters appeared on the flat surface (Figure 3b). In particular, the P(FuPy_1_-*co*-EDOT_9_) film, which possessed a higher proportion of EDOT units, revealed a loose and porous mesh-like structure, as shown in Figure 3c. Moreover, as shown in Figure 3d, a more loose and porous morphology was observed for the PEDOT homopolymer film compared to the P(FuPy_1_-*co*-EDOT_9_) copolymer film. The above analyses indicate that with the increase in EDOT units in the polymers, the polymer films show a gradual transition from the densely packed flat structure to the loose and porous network-like structure. It is noted that this porous structure may provide a large specific surface area, facilitating the rapid transport of electrolyte ions [45]. Moreover, energy-dispersive X-ray spectroscopy (EDS) was employed to further analyze the chemical composition of the resulting copolymers. Taking P(FuPy_2_-*co*-EDOT_8_) film as an example (Figure S4b), it is clear to see that C, N, O, S, and Cl elements were uniformly distributed on the film surface. The atomic ratio of N/S was decreased from 27.18, 19.57, to 10.15 for the copolymers P(FuPy_3_-*co*-EDOT_7_), P(FuPy_2_-*co*-EDOT_8_), and P(FuPy_1_-*co*-EDOT_9_), respectively (Table S1). This trend was relatively consistent with the deceased feeding mole ratio of FuPy/EDOT from 3/7, 2/8, to 1/9 for these copolymers. It should be noted here that the presence of C, N, O, and some other elements with a low Z (atomic number) were not accurately quantified due to the limitations of the semi-quantitative SEM-EDS technique [46,47].

### 2.4. Electrochemical Performances of the P(FuPy-co-EDOT) Copolymers

**Figure 4:**
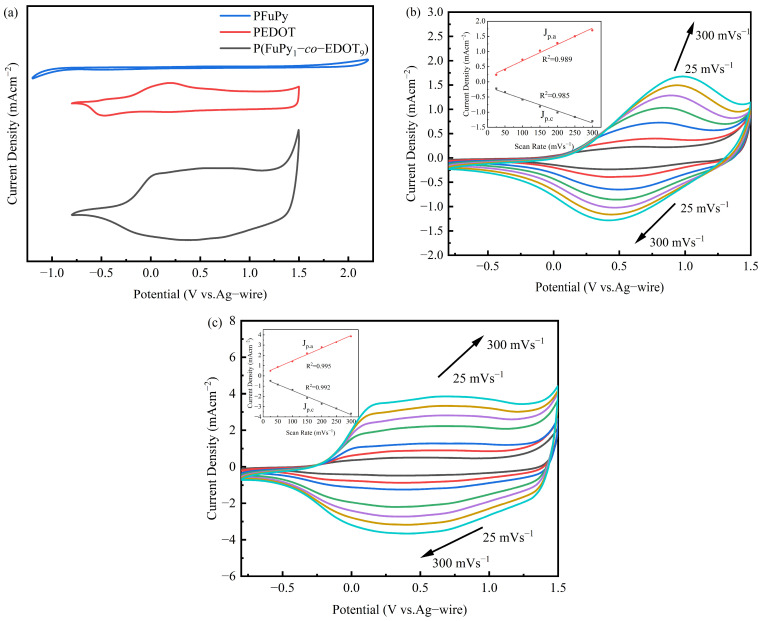
CV curves of the polymer films in 0.1 mol/L Bu_4_NClO_4_/ACN electrolyte solution: (**a**) PFuPy, PEDOT, and P(FuPy_1_-*co*-EDOT_9_) at a scan rate of 100 mV s^−1^; (**b**) P(FuPy_2_-*co*-EDOT_8_) and (**c**) P(FuPy_1_-*co*-EDOT_9_) at the scan rates ranging from 25 to 300 mV s^−1^; Inset: graph of the scan rate dependence of the anodic (*J*_p.a_) and cathodic (*J*_p.c_) peak current densities.

The electrochemical performances of the above-prepared P(FuPy-*co*-EDOT) copolymers and the related homopolymers (i.e., PFuPy, PEDOT) were investigated using cyclic voltammetry in a 0.1 mol/L Bu_4_NClO_4_/ACN electrolyte solution. As shown in Figure 4a, the PEDOT film exhibited a pair of reversible redox peaks located at 0.21 V/0.15 V and an irreversible reduction peak around −0.50 V, while the PFuPy film reflected relatively lower electrochemical redox activity, accompanied by oxidation/reduction peaks around 0.70 V/−0.53 V with low current intensities (Figure 4a and Figure S5a). This phenomenon was also found for the P(FuPy-*co*-EDOT) copolymers with a lower proportion of EDOT units, including P(FuPy_1_-*co*-EDOT_1_) and P(FuPy_3_-*co*-EDOT_7_) (Figure S5b,c). Comparably, the P(FuPy_2_-*co*-EDOT_8_) copolymers with higher ratio of EDOT units presented a couple of distinct reversible redox peaks located around 0.50 V and 0.75 V, respectively (Figure 4b). Similar pronounced redox behavior was also found for the P(FuPy_1_-*co*-EDOT_9_) copolymers (Figure 4c). A possible explanation for these features is that the presence of bulky furan side groups along the PFuPy conjugated main chain may lead to a reduction in the coplanarity and conjugation degree of the polymers due to the steric hindrance effect. Therefore, with the aid of the enforced planarity of the introduced EDOT units into the PFuPy chains, the coplanarity of the PFuPy chains may be significantly enhanced, thereby improving their effective conjugated length and corresponding to an enhanced electrochemical activity. In addition, the noticeable differences in the CV curves of the both P(FuPy_1_-*co*-EDOT_9_) copolymers and PEDOT (or PFuPy) homopolymers further indicated the formation of a copolymer, not a blend.

Moreover, the anodic (J_p.a_) and cathodic (J_p.c_) peak current densities of the P(FuPy_2_-*co*-EDOT_8_) and P(FuPy_1_-*co*-EDOT_9_) copolymer films increased linearly with the increment of scan rates from 25 to 300 mV s^−1^ (Figure 4b,c), along with a higher linear regression coefficient of R^2^ > 0.98, further suggesting the non-diffusion−controlled redox process of the resulting copolymers. This also implies that the resulting copolymer films exhibit strong adhesion to the working electrode [48,49].

### 2.5. Photophysical Properties of the P(FuPy-co-EDOT) Copolymers

**Figure 5:**
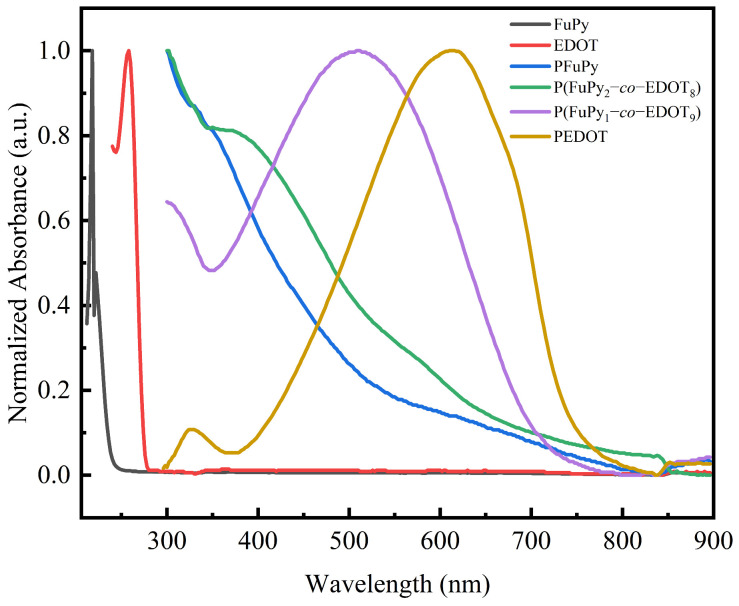
UV-Vis spectra of the FuPy and EDOT monomers in ACN solution, the dedoped homopolymer films of PFuPy and PEDOT, and the copolymer films of P(FuPy-*co*-EDOT) with different feed ratios.

The photophysical characteristics of the P(FuPy-*co*-EDOT) copolymer films were characterized using UV-Vis spectroscopy. A comparative analysis was also conducted with the spectra of the FuPy and EDOT monomers, as shown in Figure 5. The FuPy monomer exhibited double absorption peaks (*λ*_max_) at 218 nm and 222 nm, while the EDOT monomer displayed a characteristic absorption peak at 258 nm. The maximum absorption peak of the reduced homopolymer film PFuPy showed a red shift to 348 nm as compared to the FuPy monomer, indicating an increase in conjugation length of the polymer chains.

As for the copolymers, the *λ*_max_ of the dedoped P(FuPy-*co*-EDOT) films exhibited a continuous red shift from 355 nm to 510 nm as the feed ratio of FuPy/EDOT increased from 1/1 to 1/9 (Figure 5 and Figure S6, Table 1). Compared with the PFuPy homopolymer films, the *λ*_max_ of these P(FuPy-*co*-EDOT) film shows a red shift of 7~162 nm, indicating the increased effective conjugation length within the copolymer chains. Particularly, the P(FuPy_1_-*co*-EDOT_9_) copolymer film with more EDOT units demonstrated a distinct broad absorption band in the range of 350~700 nm with a center at 510 nm, which is very different to that of the P(FuPy_1_-*co*-EDOT_1_) to P(FuPy_2_-*co*-EDOT_8_), while the homopolymer PEDOT exhibited a further red-shifted *λ*_max_ at 613 nm. These phenomena indicate that the introduction of more EDOT units could significantly enhance the conjugation strength of the polymers. This occurrence is presumably due to the existence of steric hindrance effect from the furanyl groups, which may disrupt the conjugation effect of the PFuPy homopolymer chains. Adding the electron-donating EDOT to the conjugated backbones of PFuPy could greatly improve the coplanarity and enhance electronic delocalization of the conjugated chains through intramolecular charge transfer. Additionally, in comparison with the previously reported PNMPy polymer (a *N*-methyl substituted polypyrrole derivative) with a *λ*_max_ at 425 nm [36], the maximum absorption of the PFuPy polymer exhibited a 77 nm blue shift, which is probably due to the existence of the steric hindrance effect, as discussed above. In addition, the homopolymer and copolymer films were further tested under natural light and 365 nm UV light irradiation (Figure S7a). The results indicated that none of the polymer films exhibited a significant luminescent phenomenon. Furthermore, the fluorescence spectra of the polymer films were also analyzed, revealing no typical single-peak emission pattern but a multipeak-like emission feature within the range of 400 to 680 nm (Figure S7b). Interestingly, as the proportion of EDOT units in the copolymers increased, the intensity of the multipeak-like emission spectra of the polymer films was further enhanced.

Moreover, the optical band gaps (*E*_g_) of the resulting P(FuPy-*co*-EDOT) copolymers determined from their onset absorption wavelengths (*λ*_onset_) were found to be 1.75~2.02 eV, which followed the same decreasing trend with the increase in the EDOT proportions. In addition, the *E*_g_ values of the P(FuPy-*co*-EDOT) copolymers were lower than that of the PFuPy (2.58 eV) and a little higher as compared to PEDOT (1.68 eV). These features further imply that the introduction of EDOT units in the polymer backbones is beneficial for the enhancement of the conjugation extent of the polymers, generating copolymers with reduced optical bandgaps. The onset potentials can be used to determine the LUMO and HOMO energy levels of the polymers. The energy levels (in electronvolts, eV) corresponding to the electrochemical potentials (vs. SCE) can be obtained by adding 4.4 V to the potentials. Thus, *E*_HOMO_ = −e(*E*_onset_ + 4.4), where *E*_HOMO_ is the HOMO energy level and all electrode potential values are vs. SCE as the reference electrode [50,51,52]. In our measurements, the electrode potential of the Ag wire reference electrode was calibrated to be 0.004 V vs. SCE. Thus, in this work, *E*_HOMO_ = −e(*E*_onset_ + 4.404). Furthermore, the onset oxidation potentials (*E*_onset_) and HOMO/LUMO values of the monomers as well as the corresponding polymers were calculated and are summarized in Table 1. It was noted that the spectroelectrochemical data of the resulting PEDOT polymers (e.g., *λ*_max_, HOMO/LUMO energy levels, and *E*_g_) were relatively consistent with the previously reported PEDOT polymers [53], as given in parentheses in Table 1. The above studies found that by finely tuning the feed ratio of the FuPy/EDOT comonomers, a series of P(FuPy-*co*-EDOT) copolymers with lower and tunable optical band gaps can be obtained, exhibiting favorable optical absorption properties.

### 2.6. Density Functional Theory Calculations

**Figure 6:**
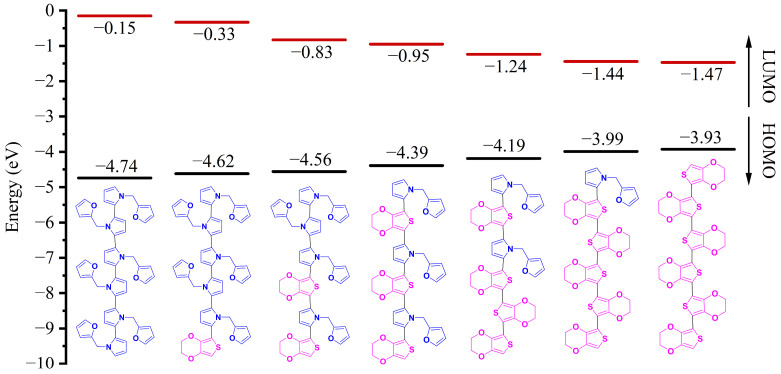
DFT-calculated HOMO and LUMO energy levels of the FuPy hexamer, FuPy/EDOT co-oligomers, and EDOT hexamer models (unit: eV).

In order to further elucidate the properties and structural characteristics of the copolymers, density functional theory (DFT) calculations were conducted on a series of model hexamers with varying compositions of FuPy and EDOT units. The optimized geometries and frontier molecular orbitals of these model hexamers were calculated using DFT on a B3LYP/6–31g(d) level, as presented in Figure S8. The calculated results for the highest occupied molecular orbital (HOMO) and the lowest unoccupied molecular orbital (LUMO) of both the optimized model hexamers are presented in Figure 6.

The HOMO orbitals of the FuPy hexamer demonstrated considerable delocalization across the entire pyrrole backbone, characterized by a predominantly delocalized π-electron distribution, with some localization observed within the furanyl groups. Notably, the substantial twisting between adjacent FuPy units significantly influenced the conjugation along the hexamer chain. Furthermore, the FuPy hexamer exhibited a lower HOMO energy level and a higher LUMO energy level compared to its EDOT counterparts. The systematic substitution of FuPy units with EDOT units in the hexamer resulted in an increasing delocalization of the HOMO and LUMO orbitals in the resulting copolymer. This delocalization extended not only along the thiophene and pyrrole backbone but also significantly over the ethylenedioxy side groups attached to the thiophene units. As the proportion of EDOT units in the oligomer increased, there was a notable enhancement in the coplanarity of the overall molecular backbone, which facilitated a greater degree of electron delocalization. Consequently, while the HOMO energy level increased, the LUMO energy level decreased. The hexamer composed entirely of EDOT units exhibited a fully planar configuration, facilitating complete electronic delocalization of the HOMO and LUMO. This corresponded to the lowest LUMO energy level observed of −1.47 eV and the highest HOMO energy level at −3.93 eV.

Additionally, the bandgap, defined as the energy difference between the HOMO and LUMO energy levels, exhibited a reduction from 4.59 eV in the FuPy hexamer to 4.29~2.55 eV in the FuPy/EDOT co-oligomers, and ultimately to 2.46 eV in the EDOT hexamer. These trends determined from DFT calculations are substantiated by experimental spectroelectrochemical data. The alignment between theoretical predictions and experimental results highlights the effectiveness of the copolymerization approach in optimizing and tailoring the optoelectronic properties of these polymers.

### 2.7. Spectroelectrochemical Performances of the P(FuPy-co-EDOT) Copolymers

**Figure 7:**
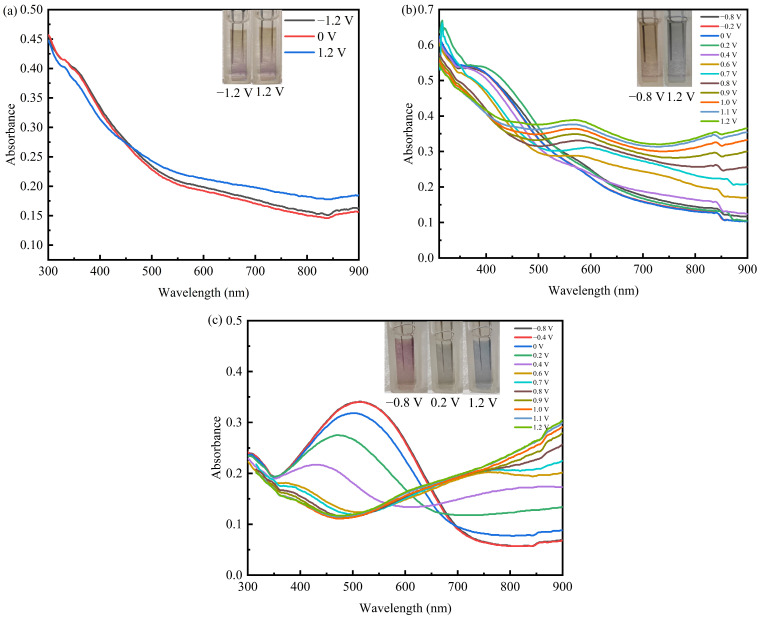
UV-Vis absorption spectra and color changes of the copolymer films at different voltages: (**a**) PFuPy; (**b**) P(FuPy_2_-*co*-EDOT_8_); (**c**) P(FuPy_1_-*co*-EDOT_9_).

Spectroelectrochemistry is an in situ technique that records the variation of UV-Vis absorption spectra of polymer films at different applied potentials. This analytical approach is used to investigate the electrochromic effect of polymer films under different doping states. The spectroelectrochemical performances of the homopolymer PFuPy film and the copolymer P(FuPy-*co*-EDOT) films were studied comparatively. As shown in Figure 7, as the voltage increased, there were almost no new absorption peaks emerging in the UV-Vis spectrum region of the homopolymer PFuPy film or even for the copolymer films of P(FuPy_1_-*co*-EDOT_1_) and P(FuPy_3_-*co*-EDOT_7_) (Figure S9). The films showed no significant color changes. There was hardly any evident electrochromic behavior observed for the homopolymer PFuPy film and the copolymer films of P(FuPy_1_-*co*-EDOT_1_) and P(FuPy_3_-*co*-EDOT_7_) under different applying voltages, which may be due to the poor electrochemical activity, as discussed above.

As for the copolymer P(FuPy_2_-*co*-EDOT_8_), shown in Figure 7b, there was a distinct absorption peak appeared at 368 nm due to the π-π* electron transition of the main chain in the reduced state (−0.8 V), resulting in a pale red appearance of the P(FuPy_2_-*co*-EDOT_8_) film. As the voltage increased, the absorption peak at 460 nm gradually diminished, while two new absorption peaks emerged at 620 nm and beyond 900 nm, resulting in a blue color finally at 1.2 V. This phenomenon indicates the formation of polarons and bipolarons under the oxidation process [10,54], while for P(FuPy_1_-*co*-EDOT_9_), the π-π* transition absorption at 514 nm gradually decreased along with other two intensified new absorptions around 760 nm and 900 nm when applying voltages from −0.8 V to 1.2 V, as depicted in Figure 7c. Meanwhile, it is very interesting to see that the P(FuPy_1_-*co*-EDOT_9_) film reflects a remarkable red-green-blue multicolor transition behavior during the oxidation process. Based on the results above, through simple adjustment of the feed ratios of FuPy and EDOT comonomers, the P(FuPy-*co*-EDOT) copolymer film with appealing multicolor electrochromic performances can be obtained.

### 2.8. Electrochromic Switching Properties of the P(FuPy-co-EDOT) Copolymer Films

In light of the aforementioned electrochromic behavior of the resulting P(FuPy-*co*-EDOT) copolymer films, further exploring the electrochromic switching performances, including optical contrast and response time, is encouraged. Optical contrast (ΔT%) and response time are two important performance evaluation indexes for electrochromic materials. ΔT% refers to the difference in visible light transmittance of the material between oxidation and reduction states, while response time is defined as the time required for color change of the material to reach 95%. Higher optical contrast and fast response time are pursued for the practical use of the electrochromic materials.

**Figure 8:**
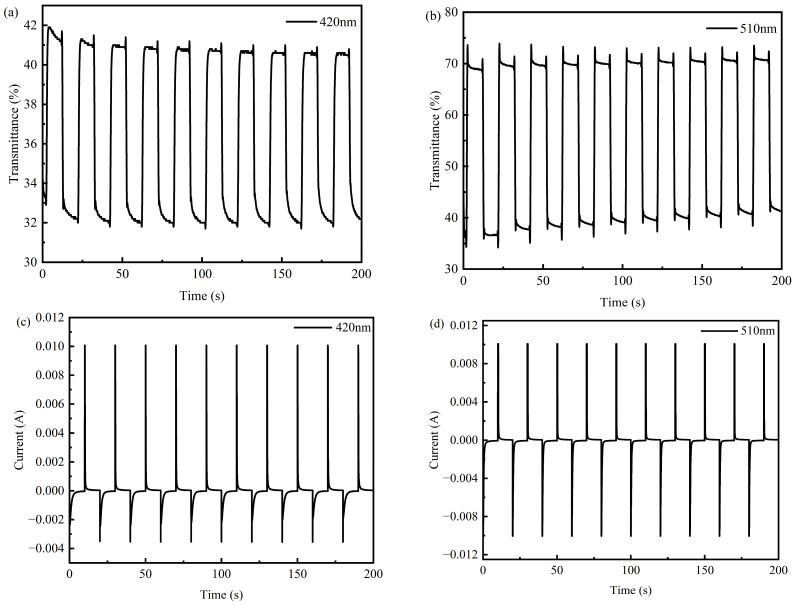
Time-dependent transmittance curves and current curve of the (**a**,**c**) P(FuPy_2_-*co*-EDOT_8_) film at 420 nm, (**b**,**d**) P(FuPy_1_-*co*-EDOT_9_) film at 510 nm between−0.8 V and 1.2 V with 10 s interval.

As shown in Figure 8, the transmittance of the polymer films at specific wavelengths was measured as a function of time by applying repeated step voltages at −0.8 V and 1.2 V with a 10 s interval. The P(FuPy_2_-*co*-EDOT_8_) copolymer film exhibited an optical contrast of 9% at 420 nm, as shown in Figure 8a. The response time for coloring (*t_c_*) and bleaching (*t_b_*) was 2.4 s and 1.3 s at 420 nm, respectively. Especially for the P(FuPy_1_-*co*-EDOT_9_) copolymer films, the optical contrast was significantly enhanced to be 34% at 510 nm with fast coloring time of 1.4 s and bleaching time of 0.8 s (Figure 8b). This represents an optical contrast improvement of approximately 25%, as compared to the P(FuPy_2_-*co*-EDOT_8_) copolymer, which may primarily be attributed to the higher doping levels of the copolymer P(FuPy_1_-*co*-EDOT_9_) with more EDOT units at the specific wavelengths.

In addition, as another key evaluation index for the electrochromic materials, the coloration efficiency (CE) was also calculated using the following formula [21]:

CE (*η*) = ΔOD/ΔQ = log(*T_b_*/*T_c_*)/(Q/A)

where *T_b_* and *T_c_*, respectively, denote the transmittance at bleaching and coloring states, ΔOD is the change in optical density, ΔQ is the amount of injected charge per unit sample area, and *η* represents the coloration efficiency (CE). As illustrated in the chronoamperometry diagrams of the copolymer film (Figure 8c,d), the current remains stable throughout the cycling process, irrespective of the applied oxidation or reduction voltages. The injected charge value can be determined by integrating the chronoamperometry curve. By calculation, the CE values were 110 cm^2^ C^−1^ at 420 nm for the P(FuPy_2_-*co*-EDOT_8_) copolymer film, and 362 cm^2^ C^−1^ at 510 nm for the P(FuPy_1_-*co*-EDOT_9_) copolymer film. According to these analyses, considering the higher optical contrast, more rapid coloring/bleaching time, and the enhanced coloration efficiency, the performance of P(FuPy_1_-*co*-EDOT_9_) copolymer film is significantly superior to that of the P(FuPy_2_-*co*-EDOT_8_) film. As tabulated in Table S2, the optical contrast of the P(FuPy_1_-*co*-EDOT_9_) copolymer film was higher than that of previous reported P(TPhSNS_1_-*co*-EDOT_4_) (18%), P(FPT-*co*-DTC) (10.6%), and P(FPT-*co*-DTP) (30%) based copolymer films. The coloring time of the copolymer films in this work was comparatively faster than that of the reported P(FPT-*co*-DTC) (2.41 s) and P(FPT-*co*-DTP) (1.78 s) copolymer films, and slightly slower than the P(ThPyDO-*co*-EDOT) (1.0 s) and P(TPhSNS_1_-*co*-EDOT_4_) (0.68 s) copolymer films. Furthermore, the coloration efficiency value of P(FuPy_1_-*co*-EDOT_9_) was higher than that of the P(ThPyDO-*co*-EDOT) (173 cm^2^ C^−1^) copolymer film [29,55,56].

### 2.9. Spectroelectrochemical Properties of the P(FuPy-co-EDOT)-Based ECDs

**Figure 9:**
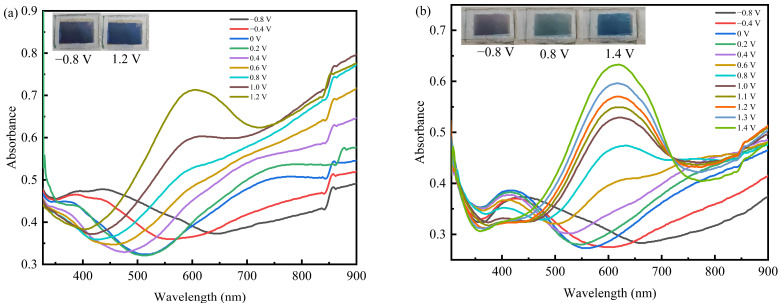
UV-Vis spectra and color changes of the P(FuPy_2_-*co*-EDOT_8_)/PEDOT (**a**) and P(FuPy_1_-*co*-EDOT_9_)/PEDOT (**b**) ECDs under different applied voltages.

In order to further explore the electrochromic properties of the resulting copolymer films within the devices, the complementary bilayer electrochromic devices (ECDs) were constructed utilizing P(FuPy-*co*-EDOT) and PEDOT as the electrochromic and counter electrode layers, respectively. For the P(FuPy_2_-*co*-EDOT_8_)/PEDOT ECDs as illustrated in Figure 8a, when applying a reduction voltage at −0.8 V, the P(FuPy_2_-*co*-EDOT_8_) layer remained in its dedoped state, exhibiting an absorption peak at 420 nm that corresponds to the π−π* electronic transition of the copolymer film. Simultaneously, the PEDOT-based counter electrode layer showed a transparent blue with little significant absorption in the UV-Vis spectrum. Considering the light colors of the both P(FuPy_2_-*co*-EDOT_8_) (pale red) and PEDOT (transparent blue) layers in this state simultaneously, the P(FuPy_2_-*co*-EDOT_8_)/PEDOT ECDs represented an overlay pale-purple-like color appearance (inset of Figure 9a). As the voltage increased, the P(FuPy_2_-*co*-EDOT_8_) layer underwent gradual oxidation, resulting in a continuous decrease in the intensity of the π-π* absorption at 420 nm. Simultaneously, the PEDOT layer was reduced, leading to the emergence of its own π-π* transition absorption around 600 nm. Ultimately, the P(FuPy_2_-*co*-EDOT_8_)/PEDOT ECDs showed a distinct blue color at 1.4 V. Furthermore, it is noteworthy that the P(FuPy_1_-*co*-EDOT_9_)/PEDOT ECDs underwent a similar spectral variation trend, as shown in Figure 8b, exhibiting a gradually weakened absorption at 436 nm and a progressively emerged and strengthened absorption around 620 nm upon stepwise oxidation. As expected, a distinct red-green-blue multicolor transition behavior was clearly observed for the P(FuPy_1_-*co*-EDOT_9_)/PEDOT ECDs when driving potentials between −0.8 V and 1.4 V, as illustrated in Figure 9b.

### 2.10. Electrochromic Switching Tests of the P(FuPy-co-EDOT)-Based ECDs

**Figure 10:**
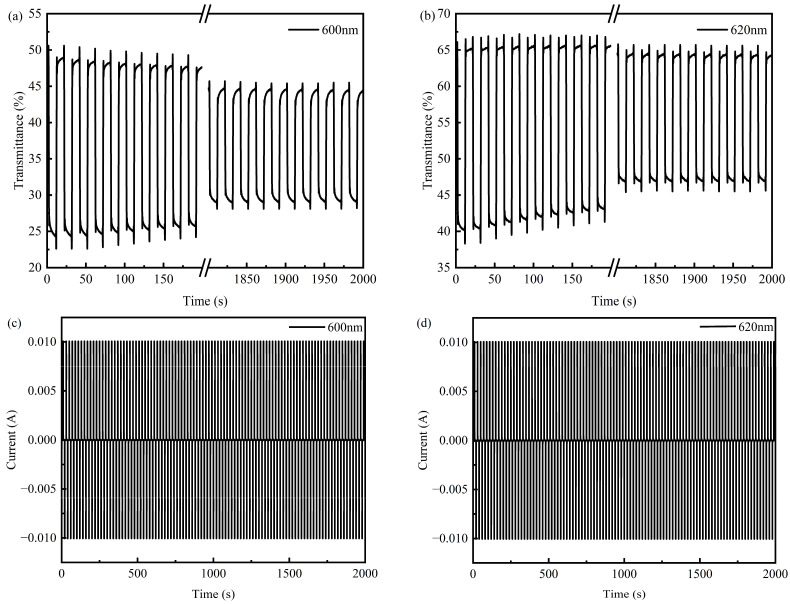
Electrochromic switching tests and current curve of the ECDs: (**a**,**c**) P(FuPy_2_-*co*-EDOT_8_)/PEDOT ECD at 600 nm between −0.8 V and 1.2 V with 10 s interval; (**b**,**d**) P(FuPy_1_-*co*-EDOT_9_)/PEDOT ECD at 620 nm between −0.8 V and 1.4 V with 10 s interval.

As shown in Figure 10, the electrochromic switching performances of the P(FuPy-*co*-EDOT)-based ECDs were also studied. The P(FuPy_2_-*co*-EDOT_8_)/PEDOT ECDs exhibited an optical contrast of 24.7% at 600 nm, with coloring and bleaching times of 0.9 s and 0.8 s, respectively (Figure 10a). After 2000 s of color change, the optical contrast decreased to 15.5%, which is approximately 63% of the original contrast, with minimal variation in response time. In comparison, the P(FuPy_1_-*co*-EDOT_9_)/PEDOT devices presented an optical contrast of about 25% at 620 nm, with the same coloring and bleaching times of 0.9 s (Figure 10b). When undergoing 2000 s of color switching, the optical contrast of the P(FuPy_1_-*co*-EDOT_9_)/PEDOT devices decreased by only 7.5%, maintaining 70% of their original transmittance contrast. Even after nearly 20,000 s (1000 cycles) of long-term cycling, these two ECDs can still maintain an optical contrast of 11~15%, which is 44~61% of their original contrast. This implies that the two ECDs have reasonable electrochromic stability (Figure S11). In addition, as shown in Figure 10c,d, the chronoamperometry curves of the two ECDs displayed significant stability during the switching process. The coloration efficiency values were calculated to be 254 cm^2^ C^−1^ at 600 nm for the P(FuPy_2_-*co*-EDOT_8_)/PEDOT devices and 416 cm^2^ C^−1^ at 620 nm for the P(FuPy_1_-*co*-EDOT_9_)/PEDOT devices. Thus, it is evident that the P(FuPy_1_-*co*-EDOT_9_)/PEDOT devices demonstrate superior optical contrast and coloration efficiency compared to the P(FuPy_2_-*co*-EDOT_8_)/PEDOT devices, which are also comparable to those polypyrroles- and polythiophenes-based electrochromic devices [29,36,57]. As illustrated in Table S3, the optical contrasts of P(FuPy_2_-*co*-EDOT_8_)/PEDOT and P(FuPy_1_-*co*-EDOT_9_)/PEDOT ECDs were higher than those of previously reported P(SNS-An-Fc)/PEDOT (15.5%), P(SNS-HE)/PEDOT (14.1%), and PNMPy/PEDOT (20.4%) based ECDs. The coloring time of the ECDs in this work was relatively faster than that of the reported P(SNS-An-Fc)/PEDOT (1.30 s) and PFPT/PProDOT-Et2 ECDs (1.01 s), and slightly slower than the PNMPy/PEDOT ECDs (0.37 s). In addition, the coloration efficiency values of the ECDs in this work were higher than that of the PNMPy/PEDOT ECDs (271 cm^2^ C^−1^), but slightly lower than those of P(SNS-An-Fc)/PEDOT (893 cm^2^ C^−1^), P(SNS-HE)/PEDOT (741 cm^2^ C^−1^), and PFPT/PProDOT-Et2 ECDs (533.5 cm^2^ C^−1^).

### 2.11. Open-Circuit Memory Capability of the P(FuPy-co-EDOT)-Based ECDs

**Figure 11:**
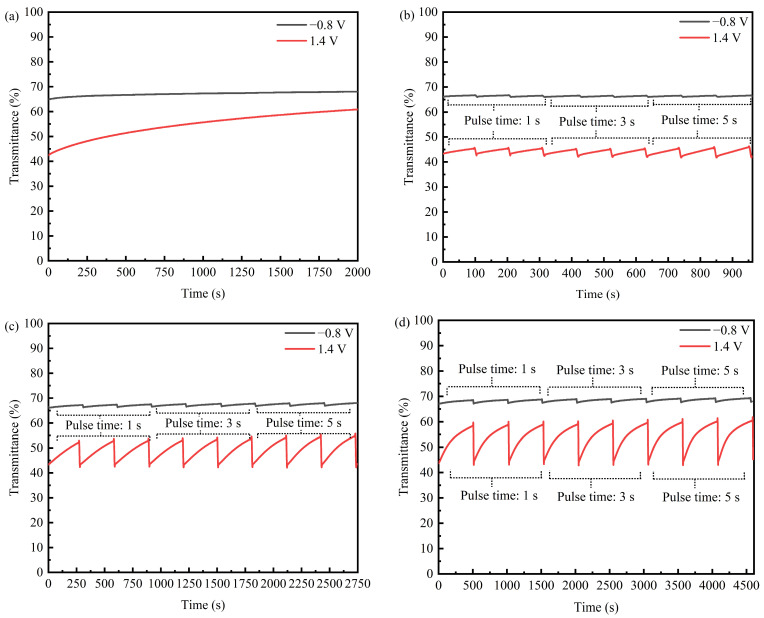
Open-circuit memory tests of the P(FuPy_1_-*co*-EDOT_9_)/PEDOT ECDs: (**a**) 2000 s under open-circuit; (**b**) 100 s of interval time; (**c**) 300 s of interval time; (**d**) 500 s of interval time.

Open-circuit memory capability is a crucial performance for electrochromic devices, as it directly influences energy consumption during operation. This parameter quantifies the ability of ECDs to maintain color under open-circuit conditions. The specific procedure involves continuously recording the transmittance curves of the ECDs over time under the individual coloring state (or bleaching state) in an open-circuit condition. During the testing process, a pulse oxidation (or reduction) voltage of 1.4 V (or −0.8 V) with a certain duration (e.g., 1, 3, and 5 s) is applied within a regular time interval (e.g., 100, 300, and 500 s). The P(FuPy_1_-*co*-EDOT_9_)/PEDOT ECDs were chosen as an example for testing. The effects of pulse voltage frequency and duration on open-circuit memory performance of the ECDs were illustrated in Figure 11. It was clear that the optical transmittance of the P(FuPy_1_-*co*-EDOT_9_)/PEDOT ECDs remained stable, with only 3% of transmittance change in the bleaching state (−0.8 V) under open-circuit condition for almost 2000 s (Figure 11a). Additionally, no significant variations in optical transmittance (0.5~1.6%) were observed at different factor level combinations (i.e., pulse voltage applied per 100, 300, and 500 s, duration time varying from 1, 3, to 5 s) in this state (Figure 11b–d, Table S4). This implies that the ECDs possess long-term open-circuit memory stability in the bleaching state. When in the coloring state (1.4 V), the transmittance of the ECDs increased gradually with time, and the color was almost completely faded after 2000 s under open-circuit conditions (Figure 11a), while this instable fading phenomenon could be significantly improved when 1 s, 3 s, or 5 s of pulse voltage (1.4 V) was applied at 100 s intervals rather than 300 and 500 s intervals (Figure 11b,d, Table S5). It is noted that the contributions of pulse voltage duration of 1 s, 3 s, and 5 s were found to be similar in minimizing transmittance variations at each pulse voltage frequency in both bleaching and coloring states. Based on these findings and in line with the principle of energy conservation, the optimal pulse voltage durations and interval times for minimizing transmittance variations should be 1 s and 2000 s in the bleaching state, and 1 s and 100 s in the coloring state. Overall, these results demonstrate that the ECDs exhibit good open-circuit memory performance, indicating their potential application in electrochromic energy-saving windows [58,59].

### 2.12. Cycling Stability of the ECDs

**Figure 12:**
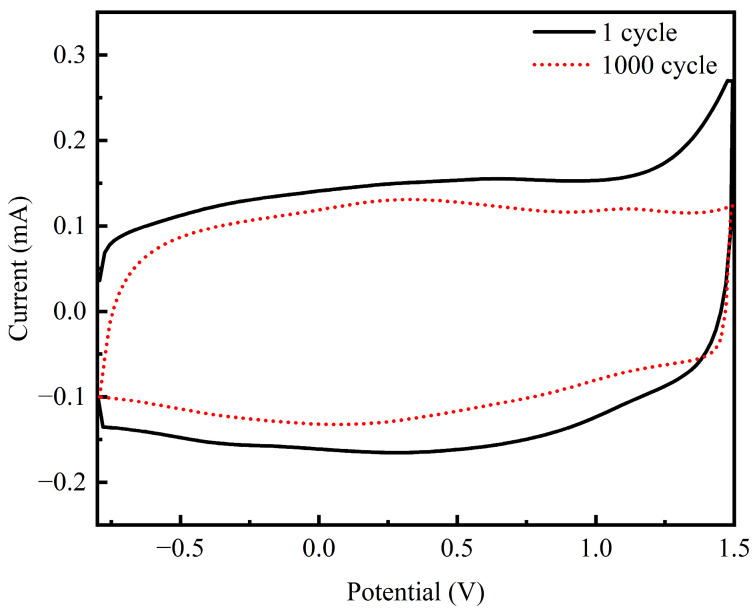
Cyclic voltammetry testing of the ECDs within a voltage range of −0.8 V to 1.4 V at a scan rate of 100 mV s^−1^.

As another essential performance index for practical application, the cycling stability was also explored via cyclic voltammetry approach to evaluate the durability of the ECDs. The P(FuPy_1_-*co*-EDOT_9_)/PEDOT ECDs were chosen as an example for testing. As illustrated in Figure 12, after 1000 cycles of testing in the driving voltage range of −0.8~1.4 V, the ECDs retained approximately 75% of their initial electroactivity, indicating their superior cycling stability. Detailed data of 1000 cyclic voltammetry cycles are shown in the supporting information (Figure S10).

## 3. Experimental

### 3.1. Materials

Acetonitrile (ACN, HPLC grade), 1-(2-furylmethyl)-1H-pyrrole (i.e., N-furfuryl pyrrole, FuPy, 98.0%), 3,4-ethylenedioxythiophene (EDOT, 98.0%), and poly(methyl methacrylate) (PMMA) were obtained from Shanghai Aladdin Reagent Co., Ltd. (Shanghai, China). Tetrabutylammonium perchlorate (Bu_4_NClO_4_, 98.0%), propylene carbonate (PC, 99.5%), and lithium perchlorate (LiClO_4_, 98.0%) were sourced from Shanghai Energy Chemical Co., Ltd. (Shanghai, China). ITO conductive glass with resistance below 10 Ω^−1^ was supplied by Shenzhen South Glass Display Devices Technology Co., Ltd. (Shenzhen, China), which was sequentially ultrasonically cleaned in ethanol, acetone, and deionized water for 10 min each, and then dried before use. The aforementioned reagents were not further purified before use.

### 3.2. Equipment

The Fourier transform infrared (FT-IR) spectra were recorded using an IR Tracer-100 infrared spectrometer supplied by Shimadzu Corporation, Kyoto, Japan. Electrochemical tests were performed with a CHI 660A electrochemical workstation (Shanghai, China). Ultraviolet-visible (UV-Vis) spectral data were obtained using a HITACHI UH4150 spectrophotometer (Hitachi, Tokyo, Japan). The surface morphology and energy-dispersive spectrometry (EDS) analyses of the polymer films were characterized using a JEOL JSM-7900F Field-emission scanning electron microscope(JEOL, Tokyo, Japan). Spectroelectrochemical data were collected using a combination of the CHI 660A electrochemical workstation (Chenhua, Shanghai, China) and the HITACHI UH4150 UV-Vis spectrophotometer (Hitachi, Tokyo, Japan).

### 3.3. Preparation of the Conducting Polymers

In this experiment, homopolymers PFuPy and PEDOT were synthesized using the electropolymerization methodology (e.g., cyclic voltammetry) employing the monomers FuPy and EDOT, respectively. Additionally, through varying the molar ratio of the comonomers FuPy and EDOT from 1:1 to 1:9, the corresponding copolymers designated as P(FuPy-*co*-EDOT) were also prepared. The polymerization was conducted in a conventional three-electrode setup, where a platinum wire or ITO conductive glass served as the working electrode, while platinum and silver wires acted as the counter and reference electrodes, respectively. The concentration of the above monomers and comonomers was all kept at 5 mmol/L using 0.1 M Bu_4_NClO_4_/ACN as the electrolyte solution. The polymerization process utilized cyclic voltammetry at a scan rate of 100 mV/s, and conducted under a nitrogen atmosphere at ambient temperature.

### 3.4. Electrochemistry Performances Tests of the Conducting Polymers

Electrochemical performances tests of the resulting conducting polymers, such as cyclic voltammetry, were performed in a three-electrode cell consisting of the polymer-coated platinum wire as the working electrode, platinum wire as the counter electrode, and Ag wire as the pseudo reference electrode. The electrolyte used was 0.1 M Bu_4_NClO_4_/ACN solution. The silver wire electrode used here was calibrated to be 0.004 V vs. SCE using ferrocene/ferrocenium (Fc/Fc^+^) redox couple in the current electrolyte system.

### 3.5. Spectroelectrochemistry Tests

Spectroelectrochemistry is a technique that combines UV-Vis spectroscopy with an electrochemistry workstation and serves as an important method for studying electrochromic properties. A three-electrode system was employed, utilizing a standard quartz cell as the electrolyte (0.1 M Bu_4_NClO_4_/ACN) container, and the ITO glass electrode coated with the polymer film served as the working electrode, while a silver wire was used as the reference electrode and a Pt wire was used as the counter electrode. When placed into a UV-visible spectrophotometer, the spectral changes of the samples were recorded in situ using different electrochemical techniques (e.g., cyclic voltammetry, chronoamperometry) under control by an electrochemical workstation.

### 3.6. Computational Details

All highest occupied molecular orbitals (HOMOs) and lowest unoccupied molecular orbitals (LUMOs) were calculated by density functional theory (DFT) calculations carried out using B3LYP/6–31g(d) within the Gaussian 16 A.03 program [60]. All calculations of geometry optimizations for the ground state (S0) and the first excited state (S1) of crystal phase were, respectively, studied using density functional theory (DFT).

### 3.7. Construction of the Electrochromic Devices (ECDs)

The classical complementary bilayer electrochromic devices (ECDs) were assembled for testing [61,62]. The polypyrrole film obtained from the aforementioned synthesis was used as the anodic coloring material, while poly(3,4-ethylenedioxythiophene) (PEDOT) served as the cathodic coloring material. A transparent gel electrolyte was prepared by mixing lithium perchlorate (LiClO_4_), acetonitrile (ACN), polymethyl methacrylate (PMMA), and propylene carbonate (PC) in a mass ratio of 3:70:7:20, followed by heating at 60 °C to form a semi-flowable gel before use. Subsequently, the gel electrolyte was uniformly applied to the surface of the polymer film, and the two films were adhered together and sealed with a light-curable glue around the edges. Finally, the obtained ECDs with active areas of about 0.8 cm × 2.0 cm were dried at room temperature and atmospheric pressure for 24 h before testing.

## 4. Conclusions

In summary, a range of novel conducting copolymers P(FuPy-*co*-EDOT) were synthesized via electropolymerization of the FuPy and EDOT comonomers. Through fine regulation of the feed ratios of FuPy/EDOT (1/1~1/9), the effects on the electrochemical, optical absorption, and electrochromic properties of the resulting copolymers were investigated in detail. The results indicated that the homopolymer PFuPy exhibited relatively low electrochemical activity and demonstrated almost no significant electrochromic behavior. When the feed ratio of FuPy to EDOT was increased to 2/8 or even 1/9, the performance of the resulting copolymer films was significantly enhanced. These films demonstrated excellent redox activity, low optical bandgaps ranging from 1.75 to 1.86 eV, and pronounced electrochromic properties. Specifically, the P(FuPy_2_-*co*-EDOT_8_) film exhibited a reversible color change from light red to blue at −0.8 V and 1.2 V, respectively, while the P(FuPy_1_-*co*-EDOT_9_) copolymer film with a higher proportion of EDOT revealed a distinct multicolor transition among red, green, and blue. Studies on the electrochromic switching performance showed that the P(FuPy_2_-*co*-EDOT_8_) film achieved an optical contrast of 9% at 420 nm, with coloring and bleaching times of 2.4 s and 1.3 s, respectively. Specially, the P(FuPy_1_-*co*-EDOT_9_) film exhibited a much higher optical contrast of 34% at 510 nm, with shortened coloring and bleaching response times of 1.4 s and 0.8 s, respectively. Furthermore, the dual-layer P(FuPy-*co*-EDOT)/PEDOT ECDs were fabricated and demonstrated exceptional optical contrast (25%) at 610 nm, rapid response time (0.9 s), and high coloring efficiency (416 cm^2^/C). The device also displayed significant open-circuit memory characteristics and long-term cycling stability. Overall, the synthesized copolymers P(FuPy-*co*-EDOT) exhibited distinctive optoelectrochemical properties, which can be precisely tailored via simple adjustment of the comonomer ratios, suggesting their extensive application potential in the field of electrochromism.

## Figures and Tables

**Table 1:** The onset oxidation potential (*E*_onset_), maximum absorption wavelength (*λ*_max_), onset absorption wavelength (*λ*_onset_), optical band gap (*E*_g_), and HOMO and LUMO energy levels values of the monomers (i.e., FuPy, EDOT), the homopolymers (i.e., PFuPy, PEDOT), and the copolymers of P(FuPy-*co*-EDOT) with different feed ratios.

Compounds	*E*_onset_, vs. (Ag-Wire) (V)	*λ*_max_/*λ*_onset_ (nm)	*E*_g_ ^a^ (eV)	*E*_HOMO_^b^ (eV)	*E*_LUMO_^c^ (eV)
FuPy	1.32	218, 222/237	5.23	−5.72	−0.49
EDOT	1.27	258/275	4.51	−5.67	−1.16
PFuPy	0.20	348/480	2.58	−4.60	−2.02
P(FuPy_1_-*co*-EDOT_1_)	0.12	355/614	2.02	−4.52	−2.50
P(FuPy_3_-*co*-EDOT_7_)	0.10	358/625	1.98	−4.50	−2.52
P(FuPy_2_-*co*-EDOT_8_)	0.03	368/667	1.86	−4.43	−2.57
P(FuPy_1_-*co*-EDOT_9_)	−0.25	510/708	1.75	−4.15	−2.40
PEDOT	−0.48 (−0.64)	613/740 (595/751)	1.68 (1.65)	−3.92 (−3.84)	−2.24 (−2.19)
PNMPy [36]	0.36	425/504	2.46	−4.84	−2.02

^a^ *E*_g_ = 1240/*λ*_onset_. ^b^ *E*_HOMO_ = −e(*E*_onset_ + 4.404) (vs. SCE). ^c^ *E*_LOMO_ = *E*_HOMO_ + *E*_g_.

## Data Availability

The data presented in this study are available on request from the corresponding author.

## Supplementary Materials

The following supporting information can be downloaded at: https://www.mdpi.com/article/10.3390/molecules30010042/s1, Figure S1: CV curves of the mixtures of FuPy/EDOT with molar ratios of 1/1 (a) and 3/7 (b) in 0.1 mol/L Bu_4_NClO_4_/ACN electrolyte solution with a scan rate at 100 mV s^−1^; Figure S2: The FT-IR spectra of the FuPy and EDOT monomers, as well as the polymer films PFuPy and PEDOT; Figure S3: The SEM images of the polymer films (scale bar: 200 nm): P(FuPy_3_-*co*-EDOT_7_); Figure S4: (a) EDS spectra of the dedoped homopolymer films of PFuPy and PEDOT, and the copolymer films of P(FuPy-*co*-EDOT) with different feed ratios; (b) EDS mapping images of P(FuPy_2_-*co*-EDOT_8_) film; Figure S5: CV curves of the polymer films in 0.1 mol/L Bu_4_NClO_4_/ACN electrolyte solution: (a) PFuPy, (b) P(FuPy_1_-*co*-EDOT_1_), (c) P(FuPy_3_-*co*-EDOT_7_) at a scan rate of 100 mV s^−1^; Figure S6: UV-Vis spectra of the copolymer films of P(FuPy_1_-*co*-EDOT_1_) and P(FuPy_3_-*co*-EDOT_7_); Figure S7: (a) The homopolymer and copolymer films under natural light and 365 nm UV light irradiation; (b) Emission spectra of the dedoped homopolymer films of PFuPy and PEDOT, and the copolymer films of P(FuPy-*co*-EDOT) with different feed ratios; Figure S8: Optimized structures and frontier molecular orbitals of HOMO and LUMO calculated with DFT on a B3LYP/6–31g(d) level of the model hexamers; Figure S9: UV-Vis absorption spectra and color changes of the copolymer films at different voltages: (a) P(FuPy_1_-*co*-EDOT_1_); (b) P(FuPy_3_-*co*-EDOT_7_); (c) PEDOT; Figure S10: Cyclic voltammetry testing of the ECDs within a voltage range of −0.8 V to 1.4 V at a scan rate of 100 mV s^−1^; Figure S11: Long-term electrochromic switching tests of the ECDs: (a) P(FuPy_2_-*co*-EDOT_8_)/PEDOT ECD at 600 nm between −0.8 V and 1.2 V with 10 s interval; (b) P(FuPy_1_-*co*-EDOT_9_)/PEDOT ECD at 620 nm between −0.8 V and 1.4 V with 10 s interval; Table S1: Atomic percentage of P(FuPy-*co*-EDOT) copolymer composites obtained from EDS spectra; Table S2: Comparison of electrochromic switching parameters of P(FuPy-*co*-EDOT) and previously reported polypyrroles- and polythiophenes-based copolymer films; Table S3: Comparison of electrochromic switching parameters of P(FuPy-*co*-EDOT)/PEDOT and previously reported polypyrroles- and polythiophenes-based ECDs; Table S4: Transmittance changes of P(FuPy_1_-*co*-EDOT_9_)/PEDOT ECDs under different pulse voltage durations and interval times at −0.8 V; Table S5: Transmittance changes of P(FuPy_1_-*co*-EDOT_9_)/PEDOT ECDs under different pulse voltage durations and interval times at 1.4 V.
