# Supplementary material for: Facile Synthesis of Novel Conducting Copolymers Based on N-Furfuryl Pyrrole and 3,4-Ethylenedioxythiophene with Enhanced Optoelectrochemical Performances Towards Electrochromic Application
Source: Molecules. 2024 Dec 26;30(1):42. doi: 10.3390/molecules30010042 (PMC11721796; doi:10.3390/molecules30010042)
Supplement: Supplementary file 1 [file molecules-30-00042-s001.zip › molecules-3308765-supplementary.pdf]

## Supplementary Materials

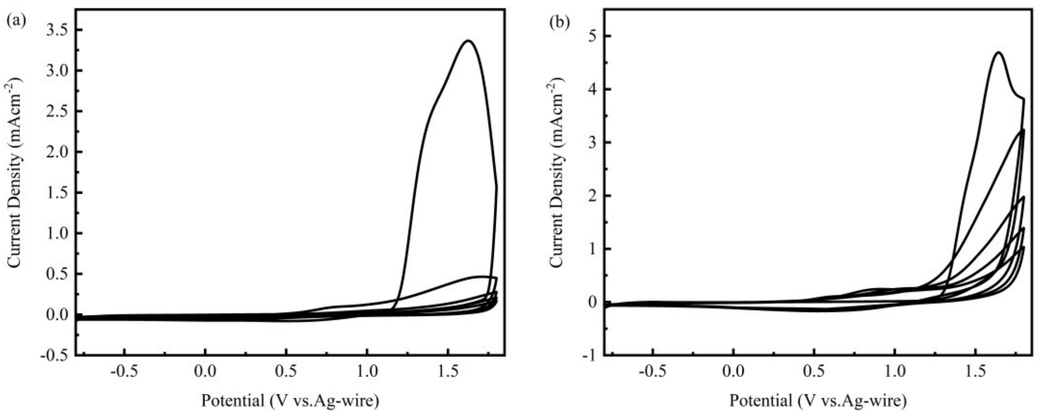


**Figure S1.** CV curves of the mixtures of FuPy/EDOT with molar ratios of 1/1 (a) and 3/7 (b) in 0.1 mol/L $Bu_4NClO_4$/ACN electrolyte solution with a scan rate at 100 mV $s^{-1}$.

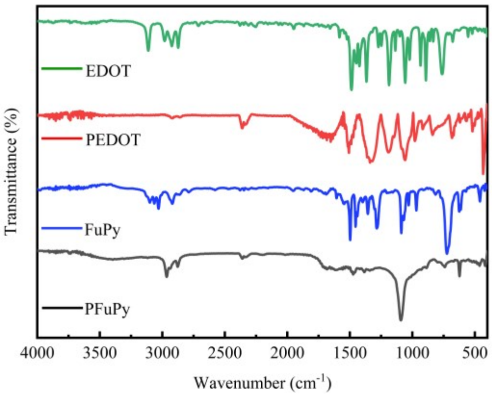


**Figure S2.** The FT-IR spectra of the FuPy and EDOT monomers, as well as the polymer films PFuPy and PEDOT.

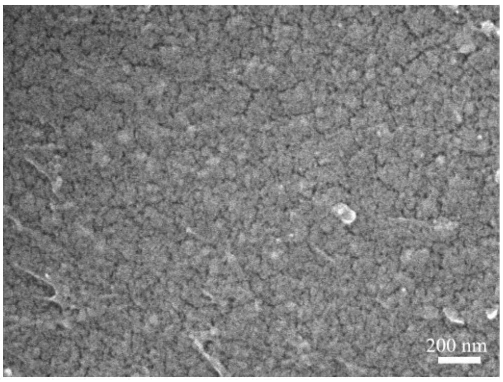


**Figure S3.** The SEM images of the polymer films (scale bar: 200 nm): P(FuPy$_3$-*co*-EDOT$_7$).

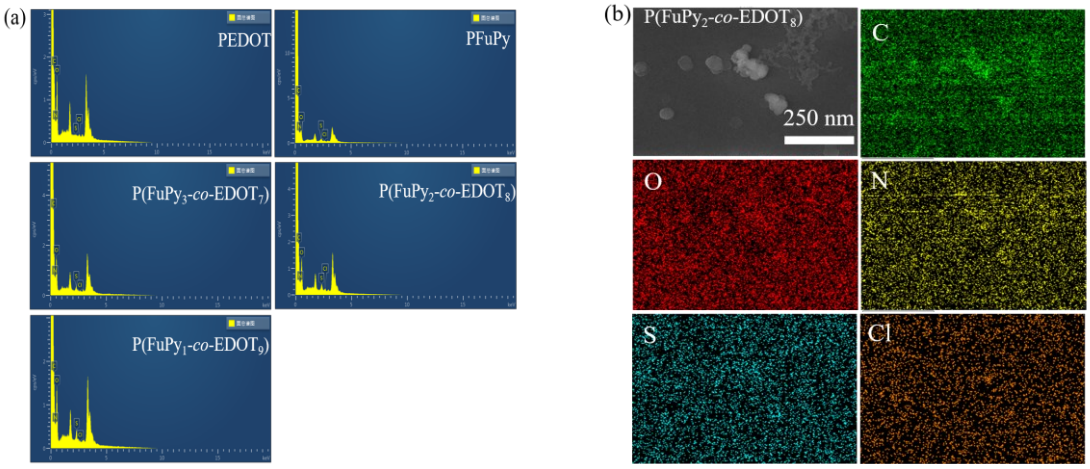


**Figure S4.** (a) EDS spectra of the dedoped homopolymer films of PFuPy and PEDOT, and the copolymer films of P(FuPy-*co*-EDOT) with different feed ratios; (b) EDS mapping images of P(FuPy$_2$-*co*-EDOT$_8$) film.

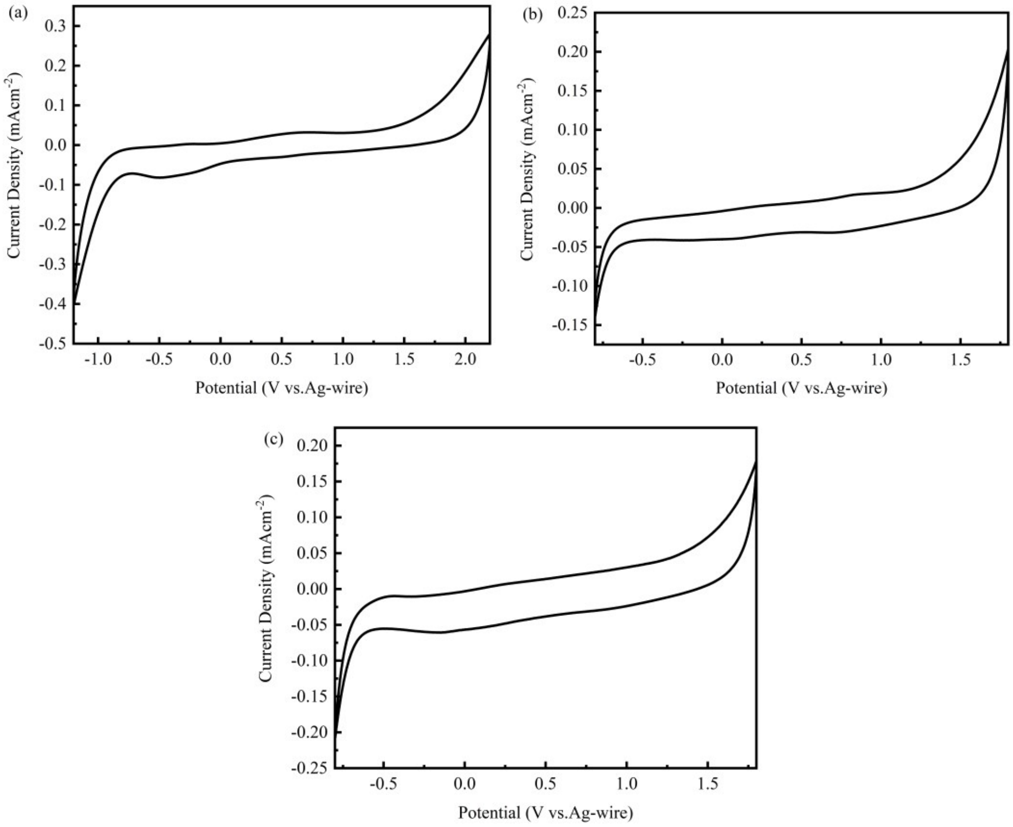


**Figure S5.** CV curves of the polymer films in 0.1 mol/L $Bu_4NClO_4$/ACN electrolyte solution: (a) PFuPy, (b) P(FuPy$_1$-*co*-EDOT$_1$), (c) P(FuPy$_3$-*co*-EDOT$_7$) at a scan rate of 100 mV $s^{-1}$.

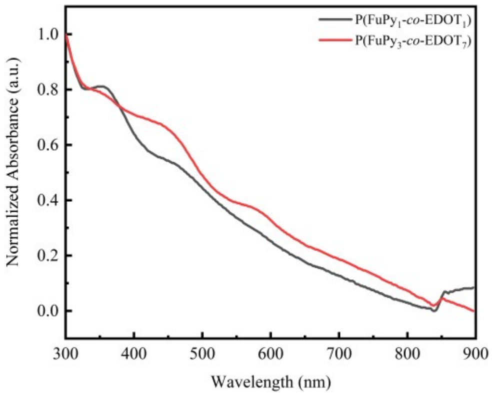


**Figure S6.** UV-Vis spectra of the copolymer films of P(FuPy$_1$-*co*-EDOT$_1$) and P(FuPy$_3$-*co*-EDOT$_7$).

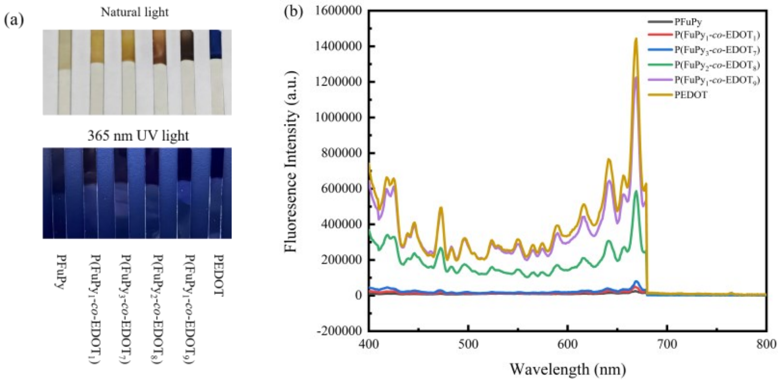


**Figure S7.** (a) The homopolymer and copolymer films under natural light and 365 nm UV light irradiation; (b) Emission spectra of the dedoped homopolymer films of PFuPy and PEDOT, and the copolymer films of P(FuPy-*co*-EDOT) with different feed ratios.

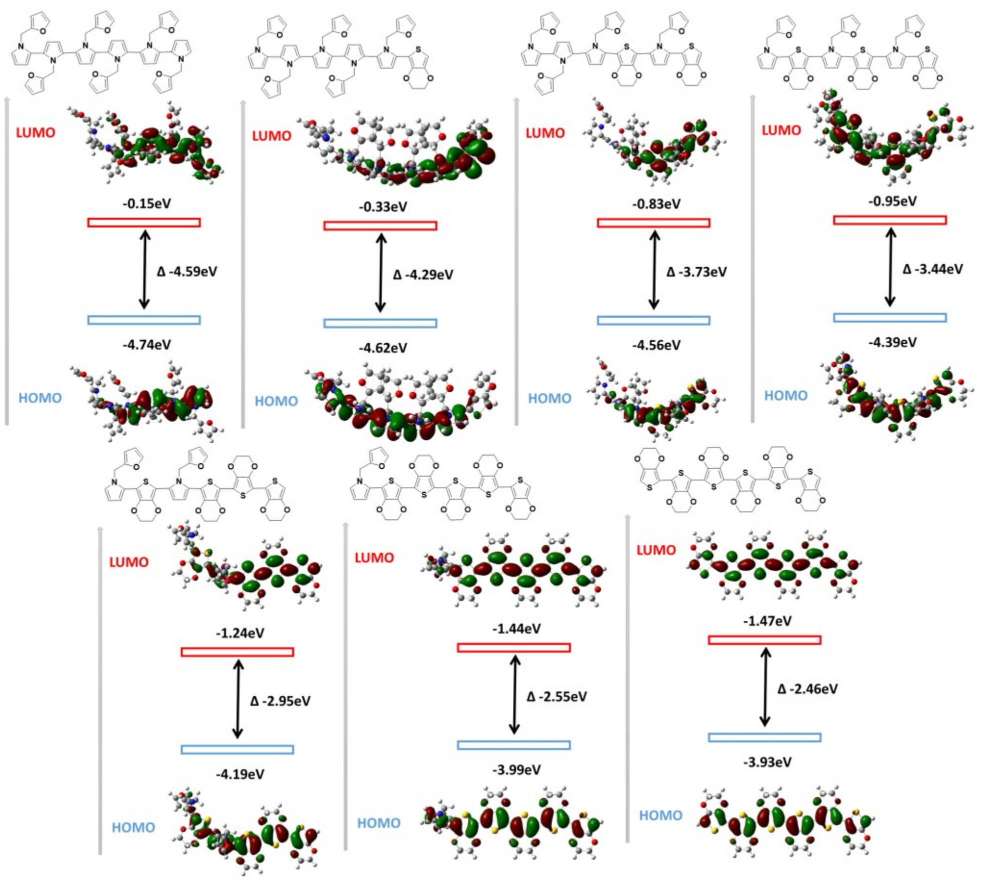


**Figure S8.** Optimized structures and frontier molecular orbitals of HOMO and LUMO calculated with DFT on a B3LYP/6-31g(d) level of the model hexamers.

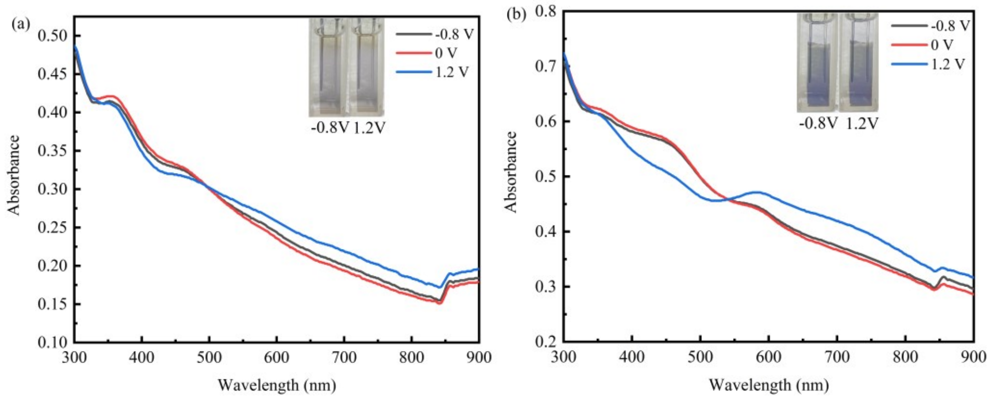

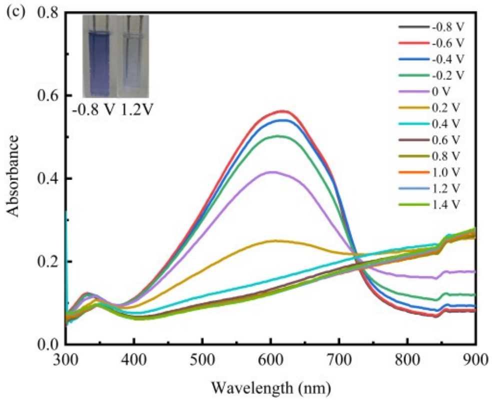


**Figure S9.** UV-Vis absorption spectra and color changes of the copolymer films at different voltages: (a) P(FuPy$_1$-*co*-EDOT$_1$); (b) P(FuPy$_3$-*co*-EDOT$_7$); (c) PEDOT.

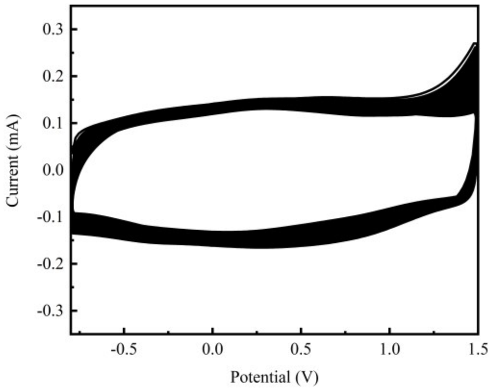


**Figure S10.** Cyclic voltammetry testing of the ECDs within a voltage range of −0.8 V to 1.4 V at a scan rate of 100 mVs$^{-1}$.

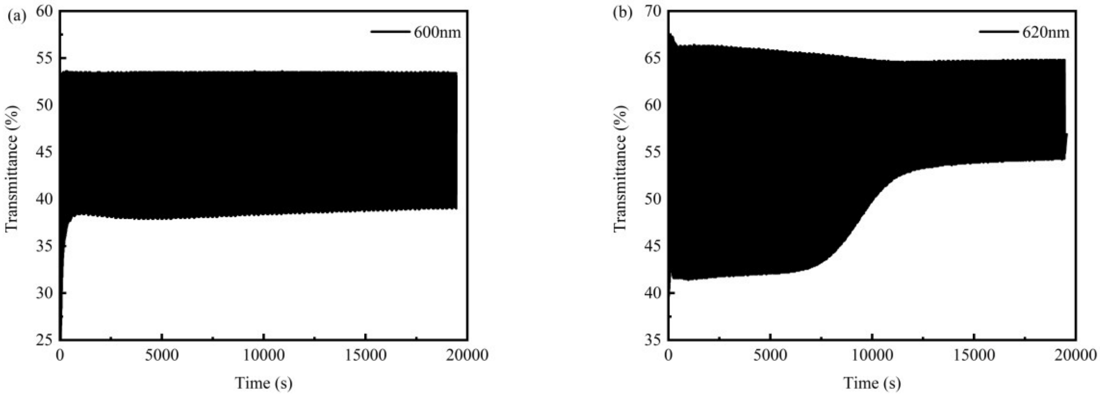


**Figure S11.** Long-term electrochromic switching tests of the ECDs: (a) P(FuPy$_2$-*co*-EDOT$_8$)/PEDOT ECD at 600 nm between −0.8 V and 1.2 V with 10

s interval; (b) P($FuPy_1$-*co*-$EDOT_9$)/PEDOT ECD at 620 nm between –0.8 V and 1.4 V with 10 s interval.

**Table S1.** Atomic percentage of P(FuPy-*co*-EDOT) copolymer composites obtained from EDS spectra

| Sample | N (atom%) | S(atom%) | N/S |
| --- | --- | --- | --- |
| PFuPy | 22.04 | 0 | - |
| PEDOT | 0 | 1.41 | - |
| P($FuPy_3$-*co*-$EDOT_7$) | 21.47 | 0.79 | 27.18 |
| P($FuPy_2$-*co*-$EDOT_8$) | 22.51 | 1.15 | 19.57 |
| P($FuPy_1$-*co*-$EDOT_9$) | 15.33 | 1.31 | 10.15 |

**Table S2.** Comparison of electrochromic switching parameters of P(FuPy-*co*-EDOT) and previous reported polypyrroles and polythiophenes based copolymer films

| Copolymer films | $\lambda_{max}$ (nm) | ΔT (%) | $t_c/t_b$ (s/s) | CE ($cm^2$ $C^{-1}$) |
| --- | --- | --- | --- | --- |
| P($FuPy_2$-*co*-$EDOT_8$) | 420 | 9 | 2.4/1.3 | 110 |
| P($FuPy_1$-*co*-$EDOT_9$) | 510 | 34 | 1.4/0.8 | 362 |
| P($TPhSNS_1$-*co*-$EDOT_4$) [57] | 436 | 18 | 0.68/0.28 | — |
| P(ThPyDO-*co*-EDOT) [58] | 500 | — | 1.0/1.0 | 173 |
| P(FPT-*co*-DTC) [29] | 406 | 10.6 | 2.41/1.95 | — |
| P(FPT-*co*-DTP) [29] | 472 | 30 | 1.78/1.97 | — |

**Table S3.** Comparison of electrochromic switching parameters of P(FuPy-*co*-EDOT)/PEDOT and previous reported polypyrroles and polythiophenes based ECDs

| ECDs | $\lambda_{max}$ (nm) | ΔT (%) | $t_c/t_b$ (s/s) | CE ($cm^2$ $C^{-1}$) |
|---|---|---|---|---|
| P($FuPy_2$-*co*-$EDOT_8$)/PEDOT | 600 | 24.7 | 0.9/0.8 | 254 |
| P($FuPy_1$-*co*-$EDOT_9$)/PEDOT | 620 | 25 | 0.9/0.9 | 416 |
| P(SNS-An-Fc)/PEDOT [59] | 610 | 15.5 | 1.30/— | 893 |
| P(SNS-HE)/PEDOT [59] | 570 | 14.1 | 0.92/— | 741 |
| PFPT/PProDOT-Et2 [29] | 590 | 33.3 | 1.01/0.89 | 533.5 |
| PNMPy/PEDOT [36] | 620 | 20.4 | 0.37/— | 271 |

**Table S4.** Transmittance changes of P($FuPy_1$-*co*-$EDOT_9$)/PEDOT ECDs under different pulse voltage durations and interval times at –0.8 V

| Pulse Voltage duration | Interval Time | | | | | | | | |
|---|---|---|---|---|---|---|---|---|---|
| | 100s | 100s | 100s | 300s | 300s | 300s | 500s | 500s | 500s |
| 1s | 0.58% | 0.69% | 0.62% | 1.21% | 1.11% | 1.21% | 1.63% | 1.58% | 1.57% |
| 3s | 0.57% | 0.55% | 0.55% | 1.19% | 1.23% | 1.23% | 1.56% | 1.58% | 1.55% |
| 5s | 0.59% | 0.55% | 0.67% | 1.26% | 1.24% | 1.16% | 1.63% | 1.65% | 1.63% |

**Table S5.** Transmittance changes of P($FuPy_1$-*co*-$EDOT_9$)/PEDOT ECDs under different pulse voltage durations and interval times at 1.4 V

| Pulse Voltage duration | Interval Time | | | | | | | | |
|---|---|---|---|---|---|---|---|---|---|
| | 100s | 100s | 100s | 300s | 300s | 300s | 500s | 500s | 500s |
| 1s | 2.1% | 2% | 2.3% | 9.28% | 9.31% | 9.1% | 13.79% | 13.46% | 13.05% |
| 3s | 2.8% | 2.96% | 3.26% | 9.26% | 9.8% | 9.85% | 13.38% | 13.84% | 13.62% |
| 5s | 3.41% | 3.99% | 4.19% | 10.58% | 10.78% | 11.07% | 14.07% | 14.2% | 14.53% |
